# Optimal Estimation of Wavelet Decomposition Level for a Matching Pursuit Algorithm

**DOI:** 10.3390/e21090843

**Published:** 2019-08-29

**Authors:** Dmitry Kaplun, Alexander Voznesenskiy, Sergei Romanov, Erivelton Nepomuceno, Denis Butusov

**Affiliations:** 1 Department of Automation and Control Processes, St. Petersburg Electrotechnical University “LETI”, Saint Petersburg 197376, Russia; 2 Control and Modelling Group (GCOM), Department of Electrical Engineering, Federal University of São João del-Rei, São João del-Rei, Minas Gerais 36307-352, Brazil; 3 Youth Research Institute, Saint Petersburg Electrotechnical University “LETI”, Saint Petersburg 197376, Russia

**Keywords:** wavelet transform, digital signal processing, spectral analysis, matching pursuit algorithm, decomposition level

## Abstract

In this paper, we consider the application of the matching pursuit algorithm (MPA) for spectral analysis of non-stationary signals. First, we estimate the approximation error and the performance time for various MPA modifications and parameters using central processor unit and graphics processing unit (GPU) to identify possible ways to improve the algorithm. Next, we propose the modifications of discrete wavelet transform (DWT) and package wavelet decomposition (PWD) for further use in MPA. We explicitly show that the optimal decomposition level, defined as a level with minimum entropy, in DWT and PWD provides the minimum approximation error and the smallest execution time when applied in MPA as a rough estimate in the case of using wavelets as basis functions (atoms). We provide an example of entropy-based estimation for optimal decomposition level in spectral analysis of seismic signals. The proposed modification of the algorithm significantly reduces its computational costs. Results of spectral analysis obtained with MPA can be used for various signal processing applications, including denoising, clustering, classification, and parameter estimation.

## 1. Introduction

Most of the real signals (seismic, biological, hydroacoustic, etc.) are non-stationary. Processing of such signals includes denoising, randomness degree estimation, short-term local features extraction, filtering, etc. Despite the fact that the processing of non-stationary signals has been studied for a long time (wavelets were described in the late 1980s), there are several unsolved problems, as follows: Working in conditions of a priori uncertainty of signal parameters, processing complex non-stationary signals with multiple local features, and multi-component signal analysis [1,2,3]. 

Current advances in applied mathematics and digital signal processing, along with the development of high-performance hardware, allow the effective application of numerous mathematical techniques, including continuous and discrete wavelet transforms. Wavelets are an effective tool for signal processing due to their adaptability, availability of fast computational algorithms, and diversity of wavelet bases.

For example, using wavelets for non-stationary signal analysis provides the following possibilities [4]:

- Detection of foreign objects, size estimation of these objects, hazard assessment based on analyzing local signal features;
- Signal detection and classification based on the analysis of local signal features;
- Signal detection in the presence of background noise; and
- Efficient signal visualization and processing based on multiscale wavelet spectrograms.

Wavelet transform uses wavelets as basis functions. An arbitrary function can be obtained from one function (“mother” wavelet) by using translations and dilations in the time domain. The wavelet transform is commonly used for analyzing non-stationary signals, usually together with various spectral analysis algorithms. 

The topic of this study is the matching pursuit algorithm (MPA), which is common technique of spectral analysis [5]. This algorithm allows decomposing the input signal using the following different basis functions: Wavelets, sine waves, damped sine waves, polynomials, etc. These functions form the atom dictionary (the set of basis functions) where each function is localized in time and frequency domains. Generally, the atom dictionary is full (all types of functions are used) and redundant (the functions are not mutually independent). One of the main problems in this technique is the selection of basis functions and dictionary optimization [6]. To solve this problem, a number of improvements and modifications of the classical algorithm were proposed by scientists.

One of the modifications of MPA was developed for geoacoustic emission signals. The geoacoustic emission signals consist of a sequence of impulses whose shape is close to the modulated Berlage functions. It is possible to use the sparse approximation method to analyze this type of signal. This method decomposes the signal into the sum of the modulated Berlage functions describing the impulses and the modulated Gaussian functions approximating the noise. Lukovenkova et al. [7] developed the adaptive modification of MPA. Its essence is the use of optimization methods for iterative refinement of the Gaussian and Berlage function parameters. In this paper, numerical methods that allow optimization of MPA and the building qualitative representations of geoacoustic signals are described.

Two of the basic prototypes of greedy optimization are abovementioned MPA and the Frank-Wolfe method. Locatello, in Reference [8], took a unified view on both methods and first calculated the explicit convergence rates of MPA in an optimization sense for general sets of atoms. Authors derive sublinear convergence for two classes on general smooth objectives and linear convergence on strongly convex objectives, as well as a clear correspondence of algorithm variants. Presented algorithms and rates are affine invariant and do not need any incoherence or sparsity assumptions. 

Marapulets et al. [9] provided the development and comparison of different numerical methods that can increase the adaptive property and improve the accuracy of MPA applied to geoacoustic and electromagnetic signals. Here, at each step of MPA, a function with the highest correlation to the original signal is chosen and the parameters of a chosen function are enhanced. The enhancement is performed by the help of different grid methods and methods based on gradient direction search. In Reference [9] the authors considered the application features of sparse approximation methods for geophysical signals of pulse nature and compared different variants of MPA modifications. 

The vibration signal measured from the mechanical equipment is associated with the operation of the key structure, such as the rolling bearing and gear. The development of effective signal processing methods for early fault detection has attracted a lot of academic attention. Such methods are of vital importance in reliability monitoring and fault diagnosis [10]. The newly proposed atomic sparse decomposition algorithm is performed around the overcomplete dictionary (instead of the traditional signal analysis method using an orthogonal basis operator). This algorithm proved its efficiency in extracting useful components from the complex signal through decreasing the influence of background noise. In Reference [10] Yong Lv et al. described an improved linear frequency-modulated function as an atom employed in the proposed enhanced orthogonal matching pursuit (EOMP) algorithm. Then, the quantum genetic algorithm (QGA) is integrated with the OMP algorithm, since the QGA can quickly obtain the global optimal solution of multiple parameters for rapidly and accurately extracting fault characteristic information from the vibration signal. The proposed method is superior to the conventional OMP algorithm in terms of accuracy and computation time. The experimental results based on the application of gear and bearing fault diagnosis indicate that it is more effective than the traditional method in extracting fault characteristic information.

The problem of overfitting arises due to the high dimension of the system, making regularization vital. Although classic regularizations provide sparsity, they fail to return highly accurate models. On the contrary, state-of-the-art group-lasso regularizations provide better results, but the cost is the low sparsity. In Reference [11], Konstantinos Skianis et al. apply a greedy variable selection algorithm, called OMP, for the text classification task. The authors also extend standard group OMP by introducing overlapping group OMP to handle overlapping groups of features. Empirical analysis examines that both OMP and overlapping GOMP constitute powerful regularizes, able to produce effective and very sparse models.

A review of relevant sources allows us to conclude that the main problems of the MPA are basis function selection and dictionary optimization. Since the choice of basis functions is made either empirically or by a priori information on the spectral composition of the signal, the focus of our study is the optimization procedures. We discuss a new way to optimize the vocabulary by automatically determining an optimal level of wavelet decomposition based on the entropy analysis. We define an optimal dictionary as a dictionary with a maximum correlation to the analyzed signal, compared to other dictionaries. We also define the optimal decomposition level as the level with minimal entropy when using DWT and PWD. When applying in MPA as a rough estimate in case of using wavelets as basis functions (atoms), the optimal decomposition level provides the minimum approximation error and the smallest execution time. 

The last of the paper is organized as follows: In Section 2 we introduce the basic matching pursuit algorithm, in Section 3 we estimate the computational complexity and approximation quality of MP, and in Section 4 we provide optimization of MPA through the estimation of the optimal level of wavelet decomposition based on the entropy value. The experimental results of the proposed algorithm compared to other algorithms and its analysis are discussed in Section 5 and Section 6, respectively. Finally, some conclusions are given in Section 7.

## 2. Basic Matching Pursuit Algorithm

Let $f\in L^{2}(R)$ be an input signal; $f=\langle f,g_{\gamma 0}\rangle g_{\gamma 0}+Rf$, where Rf is the remaining vector after the approximation in the direction $g_{\gamma 0}$. The considered problem is to minimize the remaining vector or maximize the scalar product as follows:

(1) $$Rf\to \min\Leftrightarrow \langle f,g_{\gamma 0}\rangle \to \max.$$

We search for the vector $g_{\gamma 0}$ using the following condition:

(2) $$\begin{array}{l} \left|\langle f,g_{\gamma_{0}}\rangle \right|\geq \alpha \underset{\gamma }{\sup}\left|\langle f,g_{\gamma }\rangle \right|\\ 0<\alpha \leq 1 \end{array}$$

For α=1 we obtain the classical basic matching pursuit (BMP) algorithm, otherwise-weak matching pursuit (WMP). BMP and WMP algorithms do not guarantee orthogonality of the remainder relative to the sub-space formed by the chosen atoms. This fact brings the convergence problem in the process of approximation. In order to solve this problem, one can use the orthogonal matching pursuit (OMP) algorithm [12].

The main difference between OMP and BMP is that, after every OMP step, all the coefficients extracted so far are updated by computing the orthogonal projection of the signal onto a set of currently selected atoms. This can lead to better results than BMP, but requires more computations.

The choice of vector $g_{\gamma 0}$ is not random and depends on a particular realization.

The algorithm is as follows:

- Steps n=0 ... m:
- If n=0:$R^{0}f=f$; $g_{\gamma 0}$
- Otherwise $g_{\gamma_{n}}:$
  (3) $$\left|\langle R^{n}f,g_{\gamma_{n}}\rangle \right|\geq \alpha \underset{\gamma }{\sup}\left|\langle R^{n}f,g_{\gamma }\rangle \right|,$$
  $$R^{n+1}f=R^{n}f-\langle R^{n}f,g_{\gamma_{n}}\rangle g_{\gamma_{n}}.$$
- The final decomposition is as follows [13]:
  (4) $$f=\sum_{n=0}^{m-1}\langle R^{n}f,g_{\gamma_{n}}\rangle g_{\gamma_{n}}+R^{m}f.$$

One can use several criteria [14] for stopping the algorithm, such as iterations limit, the steady error value, the absence of unused atoms, etc. Here we should note several important properties, as follows: The algorithm converges (i.e., $R^{n}\to 0$) for any *f* that is in the space spanned by the dictionary and the error $\left\|R^{n}\right\|$ decreases monotonically.

The main drawback of the MPA is its computational complexity and the main advantage is the possibility of choosing an arbitrary basis. MPA can be applied to signal, image, and video processing, shape representation and recognition, 3D objects coding, and in interdisciplinary applications like structural health monitoring [15].

## 3. A Way to Improve MPA: Reducing Computational Complexity and Approximation Quality Estimation

To estimate the computational complexity of MPA we performed the experiments using noised data with a default basis that includes several harmonic functions and wavelets. The simulation was performed on a PC under Windows 10 64-bit with CPU Intel Core i7 Skylake 4.0 GHz, RAM DDR4 Kingston HyperX Fury 64 GB 2.4 GHz, GPU NVIDIA GeForce GTX 1080 1.7 GHz DDR5 8 GB 10 GHz, and CUDA kernel 2560 with MATLAB R2018b 64-bit software.

The test signal is non-stationary, as follows:

(5) y(n)=x(n)+ε(n), n=0…N−1,

where *N* is signal length, x(n) is the Doppler curve, and ε(n) is additive white Gaussian noise.

The basis wavelet functions can be determined by a triplet (*j, n, k*), where *j* = 0 … *J* is the decomposition level (scale); $n=0\ldots 2^{j}-1$ is the node number at the current level; and $k=0\ldots \frac{N}{2^{j}}-1$ is shift value. The size of the dictionary (number of basis functions) in the case of discrete wavelet transform (DWT) or package wavelet decomposition (PWD) of level *J* is determined by the expression $M_{WP}=N\;\forall J$. The total size of the dictionary is determined as a sum of the sizes of the dictionaries included in it, as follows: $M_{DICT}=\sum_{i}M_{SUBDICT_{i}}$.

Let us estimate and compare the computational complexity of BMP and OMP algorithms. Let *H* be a Hilbert space of finite dimension *N*. A dictionary, Φ, is a set of unit norm vectors, $\varphi_{k}$, of *H* called atoms. We will also use the notation Φ for the matrix that admits the atoms, $\varphi_{k}$, as columns. A sparse approximation of a signal, *s*, over a dictionary, Φ, is a vector, *x*, with a small approximation error, ${\left\|s-\Phi x\right\|}_{2}$. Greedy methods are sub-optimal iterative algorithms that attempt to solve this problem by successively adding new atoms into a sparse approximation,$\Phi_{i}x_{i}$, with the objective of minimizing the new residual,$r_{i}=s-\Phi_{i}x_{i}$. A single iteration of the greedy algorithm consists of two successive steps [16], as follows: “Selection” and “update” (see Table 1).

The main values (Table 1) contributing to the algorithm complexity are N—the dimension of the signal space, β≥1—the redundancy of the dictionary that contains βN atoms, and *i*—the iteration number, which indicates that *i* atoms have been selected. 

**Figure 1:**
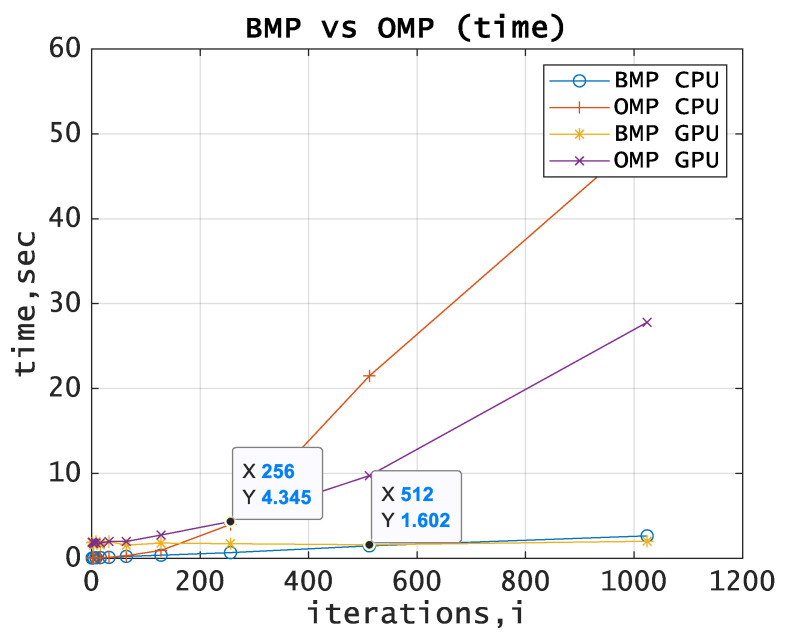
BMP vs. OMP comparison. The execution time for CPU and GPU.

Figure 1 shows the dependence between the execution time and the number of algorithm iterations. 

One can see the linear dependence between the execution time and the number of algorithm iterations for BMP and the exponential dependence for OMP. Figure 1 shows that since the number of iterations was set to 512 for BMP and 256 for OMP, the algorithm performed with GPU begins to overwhelm the algorithm performed with CPU.

**Figure 2:**
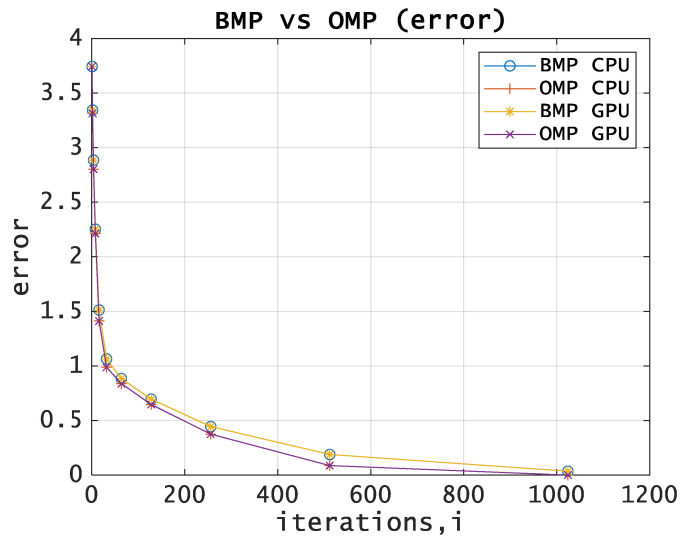
BMP vs OMP comparison. The approximation error for CPU and GPU.

Figure 2 shows the dependence between the approximation error and the number of iterations. 

One can see a slight superiority of the OMP algorithm over BMP and the exponential dependence between the approximation error and the number of iterations. 

**Figure 3:**
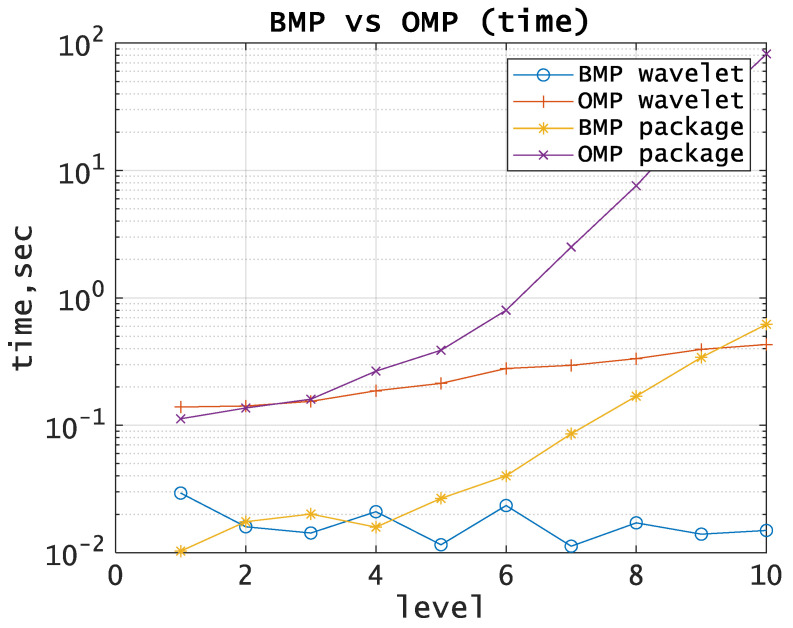
BMP vs OMP comparison. The execution time using wavelets as basis functions.

**Figure 4:**
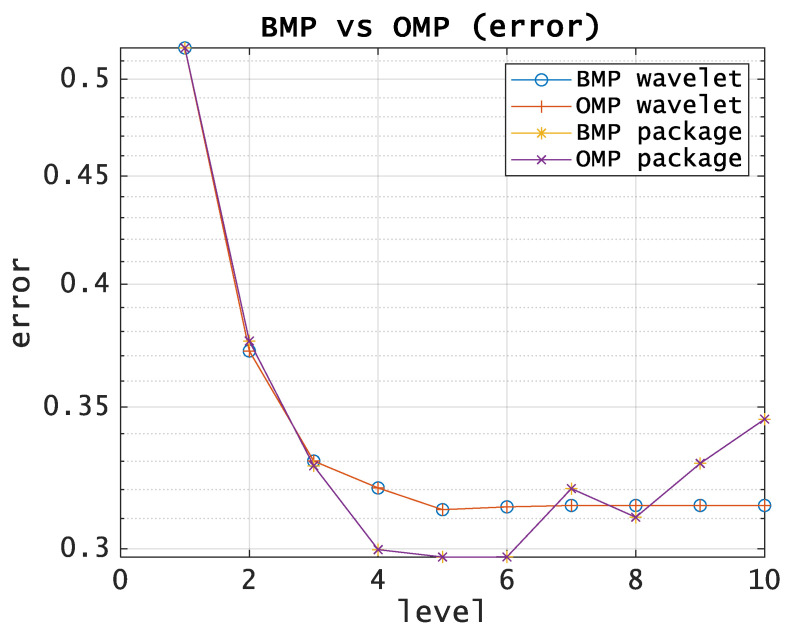
BMP vs OMP comparison. Approximation error using wavelets as basis functions.

One can see from Figure 3 and Figure 4 that the way to improve the algorithm is to optimize the decomposition level. We suppose that optimal tree provides the minimum approximation error with the smallest execution time. Application of PWD does not give a significant growth in the approximation accuracy compared to DWT and is not justified in terms of computational costs, as well as using OMP compared to BMP.

Figure 4 shows the dependence between the approximation error and the decomposition level in the case of using wavelets as basis functions.

## 4. MPA Optimization by the WT Optimal Level Estimation Based on the Entropy Criteria

A number of methods have been developed to select the (quasi) optimal tree. All of them are based on the introduction of a function (“entropy”), which allows estimation of the informativeness of a set of coefficients. The methodology is as follows. First, a complete decomposition tree is built, and then pairs of nodes with a common root are analyzed from the bottom up. If entropy does not decrease from root to node, this pair is replaced by the root. The simplified variant is to choose the optimal level, i.e., the height of the complete tree, at which the entropy is minimal [17,18].

The main problem in DWT and PWD applications is the determination of the decomposition level which determines computational costs [5]. To solve this problem, we propose the modification of these algorithms so that the optimal decomposition level is determined automatically based on the entropy value. We define the optimal decomposition level as the level with minimal entropy when using DWT and PWD. We suppose that hardware and software costs can be significantly reduced using this approach. The lower the decomposition level is, the smaller the execution time of the algorithm (Figure 3 and Figure 4).

Entropy is a common indicator characterizing the degree of uncertainty. Larger entropy values correspond to the greater randomness of the system and the smaller ones indicate the ordering of the system [17,18].

Let there be a signal, *s*, and its coefficients of expansion in some orthonormal basis, $s_{i}$. Then, the entropy,E, must satisfy the following two conditions:

1.

(6) E(0)=0,

2.

(7) $$E(s)=\sum_{i}E(s_{i}).$$

There are several known formulas for entropy, as follows:

- Shannon Entropy
  (8) $$E=-\sum_{i}s_{i}^{2}\log\left(s_{i}^{2}\right).$$
- Log Energy
  (9) $$E(s)=\sum_{i}\log\left(s_{i}^{2}\right).$$
- Threshold entropy
  (10) $$E(s)=\sum_{i:\left|s_{i}\right|>p}1,$$
  where p≥0−threshold.
- SURE
  (11) $$E(s)=n-\sum_{i:\left|s_{i}\right|\leq p}1+\sum_{i}\min(s_{i}^{2},p),$$
  where p≥0−threshold.
- Norm
  (12) $$E(s)=\sum_{i}{\left|s_{i}\right|}^{p},$$
  where p≥1 in $l^{p}$ norm.

The entropy of a function, *f*, reflects the number of essential coefficients in the expansion. If we have a set of orthonormal bases, we can choose the one that provides the lowest entropy.

We propose to do the decomposition recursively on one level for the low-frequency component (approximation coefficients) in the case of DWT and for low-frequency/high-frequency components (approximation and detail coefficients) in the case of a PWD. In each recursive call, we compare the entropy of the derived descendant nodes with the entropy of the ancestor node.

The entropy difference, as follows:

(13) $$\Delta_{i}=E_{i+1}-E_{i}.$$

Saturation condition:

(14) $$\left|\Delta_{i}\right|\leq \varepsilon .$$

Increase condition:

(15) $$\left\{ \begin{array}{l} \Delta_{i}>0\\ \left|\Delta_{i}\right|>\varepsilon \end{array}\right..$$

Decrease condition:

(16) $$\left\{ \begin{array}{l} \Delta_{i}<0\\ \left|\Delta_{i}\right|>\varepsilon \end{array}\right.,$$

where *E* denotes entropy and ε denotes accuracy.

If the entropy went into saturation (minimum is achieved), or if the limiting decomposition level is reached, decomposition stops and we thus find all the coefficients and the optimal decomposition level. The form of entropy, the limiting level, and the accuracy of the expansion are set.

## 5. Results

**Figure 5:**
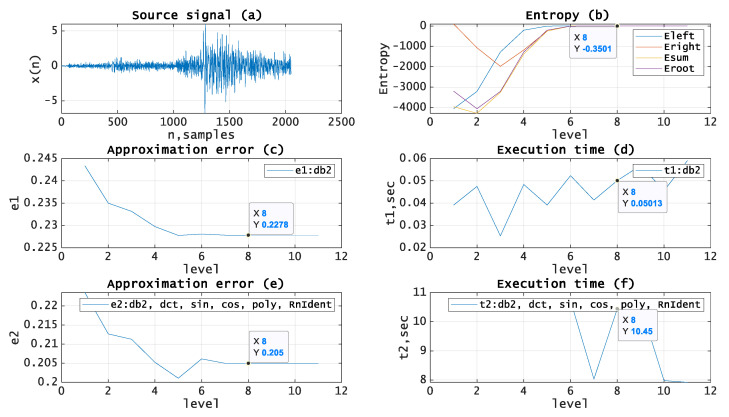
(**a**) Source signal. (**b**) Entropy (Shannon) vs. decomposition level, wavelet (Daubechies) basis. (**c**) Approximation error vs. decomposition level, wavelet (Daubechies) basis. (**d**) Execution time vs. decomposition level, wavelet (Daubechies) basis. (**e**) Approximation error vs. decomposition level, extended basis. (**f**) Execution time vs. decomposition level, extended basis.

**Figure 6:**
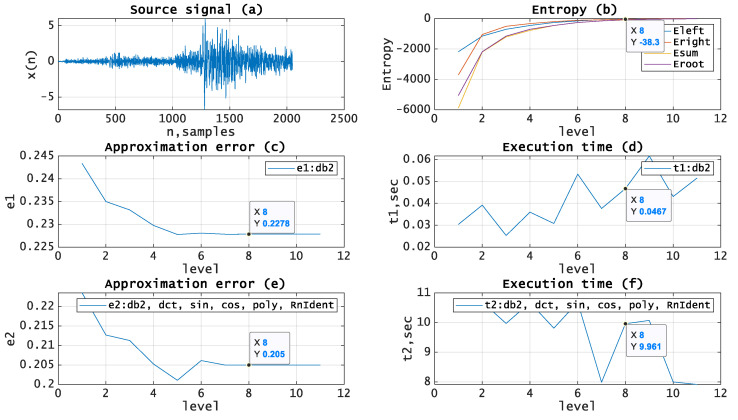
(**a**) Source signal. (**b**) Entropy (Log Energy) vs. decomposition level, wavelet (Daubechies) basis. (**c**) Approximation error vs. decomposition level, wavelet (Daubechies) basis. (**d**) Execution time vs. decomposition level, wavelet (Daubechies) basis. (**e**) Approximation error vs. decomposition level, extended basis. (**f**) Execution time vs. decomposition level, extended basis.

**Figure 7:**
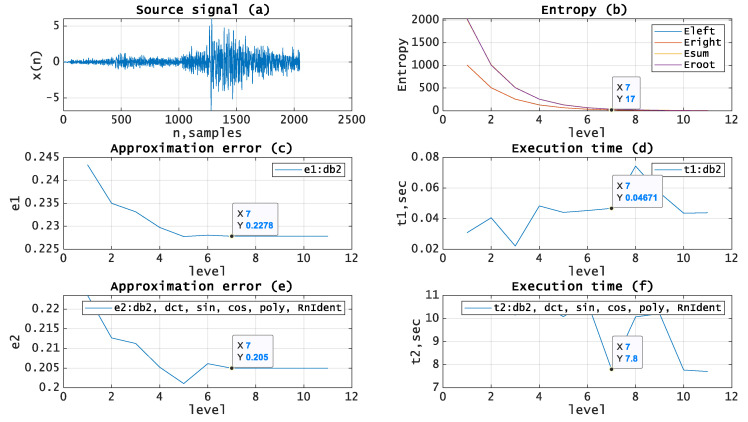
(**a**) Source signal. (**b**) Entropy (Threshold, p = 0.005) vs. decomposition level, wavelet (Daubechies) basis. (**c**) Approximation error vs. decomposition level, wavelet (Daubechies) basis. (**d**) Execution time vs. decomposition level, wavelet (Daubechies) basis. (**e**) Approximation error vs. decomposition level, extended basis. (**f**) Execution time vs. decomposition level, extended basis.

**Figure 8:**
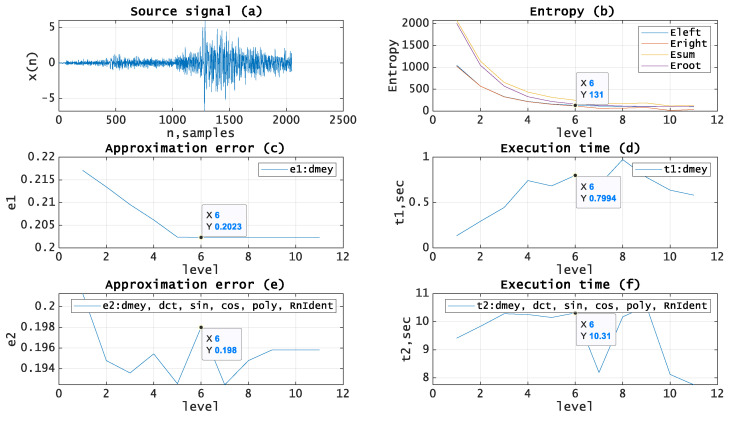
(**a**) Source signal. (**b**) Entropy (SURE, p = 0.005) vs. decomposition level, wavelet (Meyer) basis. (**c**) Approximation error vs. decomposition level, wavelet (Meyer) basis. (**d**) Execution time vs. decomposition level, wavelet (Meyer) basis. (**e**) Approximation error vs. decomposition level, extended basis. (**f**) Execution time vs. decomposition level, extended basis.

**Figure 9:**
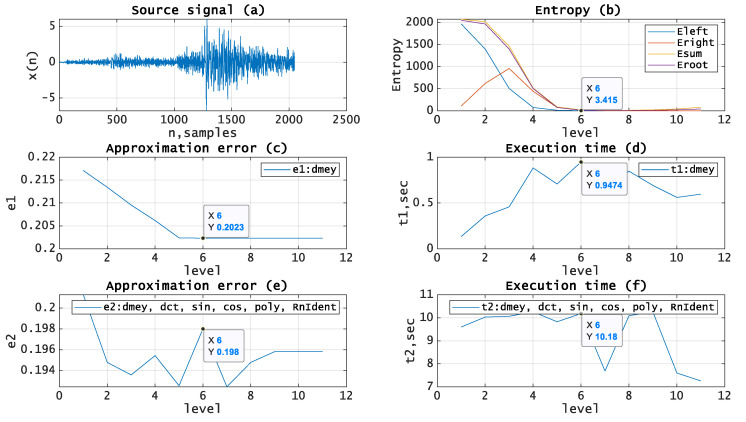
(**a**) Source signal. (**b**) Entropy (Norm, p = 2) vs. decomposition level, wavelet (Meyer) basis. (**c**) Approximation error vs. decomposition level, wavelet (Meyer) basis. (**d**) Execution time vs. decomposition level, wavelet (Meyer) basis. (**e**) Approximation error vs. decomposition level, extended basis. (**f**) Execution time vs. decomposition level, extended basis.

Let us test the given approach by applying it to the processing of real seismic signals. The experiments have been carried out using MATLAB and seismic signals from the MATLAB library [19]. Figure 5, Figure 6, Figure 7, Figure 8 and Figure 9b, depict the dependence of entropy for nodes (descendant nodes $E_{left},E_{right}$, ancestor node $E_{root}$) and the entropy sum for the two descendant nodes $E_{sum}$ (axis Y) from the decomposition level (axis X) of the DWT. The optimal level is the minimum entropy level (marked). The resulting estimation of the optimal decomposition level can be used as an approximate value in the MPA in the case of using wavelets as basis functions. Figure 5, Figure 6, Figure 7, Figure 8 and Figure 9c,e present the approximation results (axis Y-approximation error, axis X-decomposition level) using the MPA for seismic signals (Figure 5, Figure 6, Figure 7, Figure 8 and Figure 9a). Figure 5, Figure 6, Figure 7, Figure 8 and Figure 9d,f represent the performance results (axis Y-execution time, axis X-decomposition level) when using the MPA for seismic signals (Figure 5, Figure 6, Figure 7, Figure 8 and Figure 9a). We used DWT and a dictionary of atoms consisting only of wavelets (Figure 5, Figure 6, Figure 7, Figure 8 and Figure 9c,d) or wavelets and other functions from extended basis (Figure 5, Figure 6, Figure 7, Figure 8 and Figure 9e,f), e.g., harmonics, polynomials, and delta-functions. Various entropy criteria and wavelet bases were used. The approximation error was calculated using the SD formula, as follows:

(17) $$\Delta =\sqrt{\frac{1}{N}\sum_{i=1}^{N}{\left(s_{i}-{\tilde{s}}_{i}\right)}^{2}},$$

where *N* is signal length and $s,\tilde{s}$ are signal and its approximation.

Figure 5 shows the source signal (a) with length *N = 2048*, Shannon entropy curves for wavelet (Daubechies [4]) basis (b) with the estimated optimal decomposition level *X* = *8*, the approximation error for wavelet (Daubechies) basis (c) using the optimal decomposition level X = 8, the execution time for wavelet (Daubechies) basis (d) using the optimal decomposition level X = 8, the approximation error for extended basis (e) using the optimal decomposition level X = 8, and the execution time for extended basis (f) using the optimal decomposition level X = 8.

Figure 6 shows source signal (a) with length *N* = *2048*, entropy (log energy) curves for wavelet (Daubechies) basis (b) with the estimated optimal decomposition level *X* = *8*, the approximation error for wavelet (Daubechies) basis (c) using the optimal decomposition level X = 8, the execution time for wavelet (Daubechies) basis (d) using the optimal decomposition level X = 8, the approximation error for extended basis (e) using the optimal decomposition level X = 8, and the execution time for extended basis (f) using the optimal decomposition level X = 8.

Figure 7 shows source signal (a) with length *N* = *2048*, entropy (Threshold, *p* = 0.005) curves for wavelet (Daubechies) basis (b) with the estimated optimal decomposition level *X* = *7*, the approximation error for wavelet (Daubechies) basis (c) using the optimal decomposition level X = 7, the execution time for wavelet (Daubechies) basis (d) using the optimal decomposition level X = 7, the approximation error for extended basis (e) using the optimal decomposition level X = 7, and the execution time for extended basis (f) using the optimal decomposition level X = 7.

Figure 8 shows source signal (a) with length *N* = *2048*, entropy (SURE, *p* = 0.005) curves for wavelet (Meyer [4]) basis (b) with the estimated optimal decomposition level *X* = *7*, the approximation error for wavelet (Meyer) basis (c) using the optimal decomposition level X = 7, the execution time for wavelet (Meyer) basis (d) using the optimal decomposition level X = 7, the approximation error for extended basis (e), and the execution time for extended basis (f) using the optimal decomposition level X = 7.

Figure 9 shows source signal (a) with length *N* = *2048*, entropy (Norm, *p* = 2) curves for wavelet (Meyer) basis (b) with the estimated optimal decomposition level *X* = *7*, the approximation error for wavelet (Meyer) basis (c) using the optimal decomposition level X = 7, the execution time for wavelet (Meyer) basis (d) using the optimal decomposition level X = 7, the approximation error for extended basis (e) using the optimal decomposition level X = 7, and the execution time for extended basis (f) using the optimal decomposition level X = 7.

The entropy concept comes from the information theory (Shannon entropy). In the Shannon formula, probabilities are used to calculate the entropy (i.e., numbers in the range 0–1) the minus sign in front of the sum sign is to compensate for the negative value resulting from the probabilities logarithm. However, in the case of using the entropy criteria in digital signal processing, the signal samples in the general case can go beyond the range 0–1 and, therefore, the logarithm will not give a negative result, so the Shannon entropy will be negative. Depending on the use of one or another entropy criterion, we look for the minimum or maximum of its value (minimum modulus).

In Table 2, the optimal level (in terms of our paper) and basic level are presented for the proposed algorithm (optimization) as well as the maximum level decomposition without optimization that is determined by the signal length ($L_{\max}=\log_{2}N$).

From Table 2, we can see that the proposed approach in most cases can significantly reduce the decomposition level and therefore reduce the computational costs when performing MPA (Figure 5, Figure 6, Figure 7, Figure 8 and Figure 9). One can see from Figure 3, that we can estimate the approximate execution time of the algorithm and estimate the gain in execution time for the proposed method.

## 6. Discussion

There are some known approaches to construct an optimal decomposition tree based on entropy in various applications [20,21]. To optimize the packet wavelet decomposition tree in MATLAB, two optimization functions based on different entropy criteria were presented [22,23]. These functions have a number of drawbacks, as follows: Optimization is performed postfactum and tree optimization for the DWT is not considered. In this study, we proposed and experimentally investigated two modifications of the algorithms of DWT and PWD that allow for constructing the optimal tree dynamically. The proposed algorithms are based on entropy estimation. We suppose that the optimal tree provides the minimum approximation error with the smallest execution time when applied in MPA. The experimental results confirm the theoretical suggestions which depend on ε, as follows:

- Execution time depends on the decomposition basis (level, mother wavelet) (Figure 3).
- Approximation error depends on the decomposition basis (level, mother wavelet) (Figure 4).
- Using the wavelet basis provides a gain in time, but increases the approximation error (Figure 5, Figure 6, Figure 7, Figure 8 and Figure 9c–d).
- Using the extended basis reduces the approximation error, but eliminates the time gain (Figure 5, Figure 6, Figure 7, Figure 8 and Figure 9e–f).

The various results obtained for different bases and entropy criteria can be explained by the properties of the mother wavelets and the different entropy formulas. There is no straight dependence of the results of the proposed method on the choice of the type of entropy.

The proposed approach has no specificity and can be used in the processing of various non-stationary signals (hydroacoustic, biological, etc.).

## 7. Conclusions

The MPA has been studied for spectral analysis applications and its software implementation has been performed for signals with different parameters. The approximation accuracy and execution time of the algorithm were estimated using CPU and GPU platforms.

The novel modifications of DWT and PWD have been proposed for further implementation in MPA. We explicitly show that the optimal decomposition level (with minimal entropy) in DWT and PWD provides a minimal approximation error and the shortest execution time applied in MPA as a rough estimate in the case of using wavelets as basis functions.

All of the entropy curves for the chosen range of the decomposition level (the maximum level of decomposition is determined by the signal length, as follows: $L_{\max}=\log_{2}N$) are exponential. Thus, one can obtain the optimal decomposition level with minimum entropy having a given accuracy,ε. In most cases, it is possible to obtain the optimal level and it is not so high as the maximum level (Table 2), but this provides a trade-off between computational complexity and the runtime (Figure 5, Figure 6, Figure 7, Figure 8 and Figure 9).

We have provided an example of entropy-based estimation for the optimal decomposition level in spectral analysis of seismic signals. It is shown that the proposed modification of the algorithm significantly reduces its computational costs. 

## 8. Supplementary

Below we present the code of modified algorithms (DWT, PWD) in MATLAB:

function CF = wavedec_adaptive(x,wname,max_level,entropy,threshold)

%%%%%%%%%%%%%%%%%%%%%%%%%%%%%%%%%%%%%%%%%%%%%%%%%%%%

% Adaptive DWT function

% Input:

% x-signal

% wname-wavelet name

% max_level-max decomposition level

% entropy-entropy criterion

% threshold-entropy threshold

% epsilon-entropy decay rate

% Output:

% CF-decomposition coefficients (all levels)

%%%%%%%%%%%%%%%%%%%%%%%%%%%%%%%%%%%%%%%%%%%%%%%%%%%%

global level E_L E_R E_S E_RT lvl

eps = 10;

 

[A,D] = dwt(x,wname); % decomposition into 1 level

CF = [{A} {D}];    % union

level = level + 1;   % tree depth (stack)

 

E_RT (level) = wentropy(x,entropy,threshold);  % root entropy

E_L(level) = wentropy(A,entropy,threshold);   % left node entropy

E_R(level) = wentropy(D,entropy,threshold);  % right node entropy

E_S(level) = E_L(level) + E_R(level);    % total entropy

 

if level ==1

  delta = E_L(level)-E_RT(level);

else

  delta = E_L(level)-E_L(level-1);

end

 

if(level > = max_level || (delta > 0 && abs(delta) > eps) || abs(delta) < = eps) % stop criterion

  lvl = level;

  level = level-1;  % tree depth (stack)

  return;

end

 

CF = [wavedec_adaptive(A,wname,max_level,entropy,threshold) {D}]; % further decomposition

level = level-1;   % tree depth (stack)

 

end

 

 

function [CF] = wpdec_adaptive(x,wname,max_level,entropy,threshold)

 

%%%%%%%%%%%%%%%%%%%%%%%%%%%%%%%%%%%%%%%%%%%%%%%%%%%%

% Adaptive PWD function

% Input:

% x-signal

% wname-wavelet name

% max_level-max decomposition level

% entropy-entropy criterion

% threshold-entropy threshold

% epsilon-entropy decay rate

% Output:

% CF-decomposition coefficients (all levels)

%%%%%%%%%%%%%%%%%%%%%%%%%%%%%%%%%%%%%%%%%%%%%%%%%%%%

 

global level E_L E_R E_S E_RT lvl

eps = 10;

 

[A,D] = dwt(x,wname);  % decomposition into 1 level

CF = [{A} {D}];    % union

 

level = level+1;    % tree depth (stack)

 

E_RT (level) = wentropy(x,entropy,threshold);  % root entropy

E_L(level) = wentropy(A,entropy,threshold);   % left node entropy

E_R(level) = wentropy(D,entropy,threshold);  % right node entropy

E_S(level) = E_L(level) + E_R(level);    % total entropy

 

if level == 1

  delta = E_L(level)-E_RT(level);

else

  delta = E_L(level)-E_L(level-1);

end

 

if(level > = max_level || (delta > 0 && abs(delta) > eps) || abs(delta) < = eps) % stop criterion

  level = level-1;   % tree depth (stack)

  return;

end

 

CF1=wpdec_adaptive(A,wname,max_level,entropy,threshold); % further decomposition

 

if level == 1

  delta = E_R(level)-E_RT(level);

else

  delta = E_R(level)-E_R(level-1);

end

 

if(level > = max_level || (delta > 0 && abs(delta) > eps) || abs(delta) < = eps) % stop criterion

  level = level-1;   % tree depth (stack)

  return;

end

 

CF2 = wpdec_adaptive(D,wname,max_level,entropy,threshold); % further decomposition

 

CF = [CF1 CF2];

level = level-1;   % tree depth (stack)

 

end

## Figures and Tables

**Table 1:** The order of complexity of a given iteration for general cases of greedy algorithms.

	Step	BMP	OMP
selection	correlations	$\beta N^{2}$	$\beta N^{2}$
	maximum	βN	βN
update	Gram matrix	0	*iN*
	coefficients	0	*i* ^2^
	residual	*N*	*iN*

**Table 2:** Proposed algorithm (optimization, first value) vs. maximum level decomposition (no optimization, second value).

Entropy	Daubechies Basis	Meyer Basis
Shannon	8/11	6/11
Log Energy	8/11	6/11
Threshold	7/11	6/11
Sure	7/11	6/11
Norm	7/11	6/11
